# From Womb to World: Exploring the Immunological Connections between Mother and Child

**DOI:** 10.4049/immunohorizons.2400032

**Published:** 2024-08-22

**Authors:** Bobby J. Cherayil, Nitya Jain

**Affiliations:** * Mucosal Immunology and Biology Research Center, Mass General for Children, Charlestown, MA; † Department of Pediatrics, Harvard Medical School, Boston, MA; ‡ Center for Computational and Integrative Biology, Mass General Brigham, Boston, MA

## Abstract

Mother and child are immunologically interconnected by mechanisms that we are only beginning to understand. During pregnancy, multiple molecular and cellular factors of maternal origin are transferred across the placenta and influence the development and function of the fetal and newborn immune system. Altered maternal immune states arising from pregnancy-associated infections or immunizations have the potential to program offspring immune function in ways that may have long-term health consequences. In this study, we review current literature on the impact of prenatal infection and vaccination on the developing immune system, highlight knowledge gaps, and look to the horizon to envision maternal interventions that could benefit both the mother and her child.

## Introduction

The maternal immune system undergoes significant modifications during pregnancy to establish tolerance toward the semiallogeneic fetus while maintaining the ability to protect the mother and developing fetus from infections. Numerous factors, such as nutrition, stress, and hormones, regulate maternal immune status during pregnancy, and readers are directed to excellent reviews that cover these topics (1–4). For the most part, any event that impacts maternal immunity can have an influence on pregnancy itself as well as on the developing fetal immune system. The early-life immune system develops in functional layers (5). Given the developmental plasticity inherent in these early-life processes, it may be appropriate to extend the principles of the Developmental Origins of Health and Disease hypothesis (6) to the immune system. This theory posits that exposure to environmental stimuli during critical periods of prenatal development can have profound effects on an individual’s short- and long-term health. For example, adverse conditions in the uterine environment can lead to alterations in fetal endocrine and metabolic activity, resulting in altered growth and irreversible changes in the structure and function of vital organs (7–9). Subsequent environmental influences during infancy and childhood further reinforce the risk of disease later in life (10–12).

**FIGURE 1.**
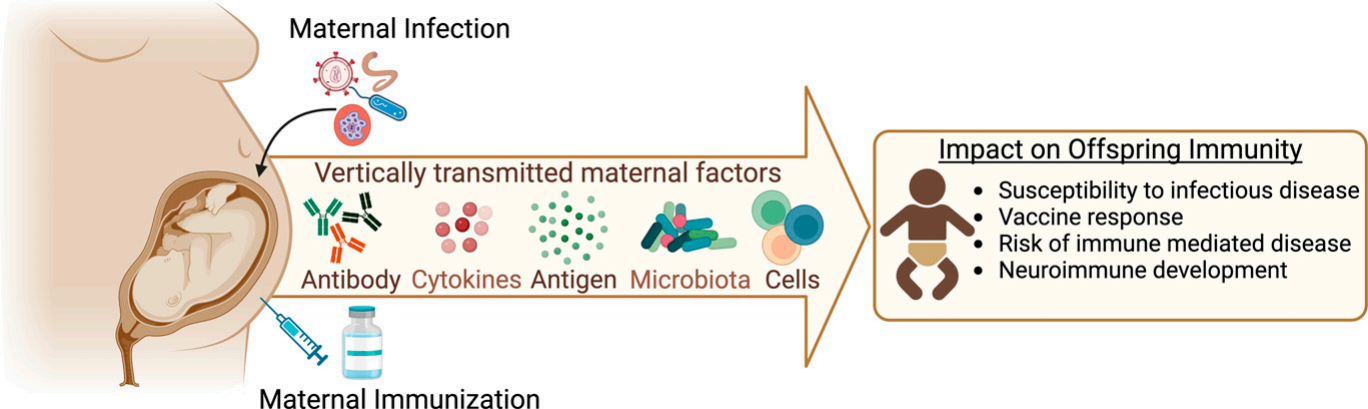
The mother–infant dyad: vertically transferred immunity. Offspring immunity is influenced by several vertically transferred maternal factors, including Ab, cytokines, Ag, microbes, and cells. Maternal immune states arising from pregnancy-associated infections and immunizations can impact these pathways and have long-term consequences on offspring health, including susceptibility to infections and immune-mediated diseases as well as neurodevelopment. The figure was created using BioRender.com.

Exposure to infectious agents during pregnancy can affect the developing fetal immune system and impact subsequent newborn immune responses to infections and vaccinations (13). Understanding the mechanisms of maternal influence on offspring immunity is crucial for formulating strategies to mitigate the long-term health impacts of adverse developmental immune imprinting. In this article, we provide an overview of the various ways in which a pregnant female can communicate immunologically with her developing offspring (Fig. 1). We focus on the impact of pregnancy-associated infections on these channels of communication and how they impact offspring immunity. Last, we discuss current maternal immunization strategies and highlight areas of uncertainty and future research priorities.

### Mechanisms mediating maternal effects on offspring immunity

Mother and offspring constitute a single interconnected unit during pregnancy and for about 1 y after birth. Through the course of this period, an array of maternal molecules and cells are transferred to the fetus and infant, with some providing passive antimicrobial protection and others influencing the development and function of the baby’s own immune defenses (14). The best studied of the molecules are maternal Abs: IgG, which is transported across the placenta into the fetal circulation and confers passive humoral immunity on the newborn infant for 3–6 mo (15), and IgA, which is conveyed to the infant through breast milk and helps in defending against intestinal infection and in shaping the gut microbiota (16). Other transplacentally transferred maternal molecules that can affect the offspring immune system include inflammatory mediators, Ags, nutrients, and microbiota-derived components and metabolites. Constituents of breast milk apart from IgA can also influence offspring immunity. They include immunomodulatory cytokines such as TGF-β and specific oligosaccharides that promote the development of a healthy gut microbiota and inhibit pathogen colonization (17, 18).

In addition to soluble molecules, maternal cells can make their way across the placenta and into breast milk (19–21). The effects of this mode of maternal–offspring interaction are not well understood, especially in humans. In mouse studies, transplacentally transferred cells from the mother have been implicated in the development of tolerance to noninherited maternal Ags in the fetus (14). Similarly, animal experiments have shown that maternal cells in breast milk can survive transit through the neonatal gut and even traffic to distal sites, but their influence on offspring immune function remains rather speculative (22).

A final mechanism by which the mother can affect the infant’s immune system is by transfer of her microbiota at the time of delivery (5). The fetus does not have a resident microbial community and is first exposed to microorganisms during birth. The newborn’s tissues are initially colonized by organisms derived from either the mother’s intestinal and genital tracts (if the delivery is vaginal) or from the mother’s skin and the hospital environment (if the delivery is by cesarean section) (23, 24). These pioneer microbes set the stage for subsequent waves of colonization until a stable, adult-type microbiota is established by the age of 2–3 y. Studies in mice and humans over the last 10–20 y have provided compelling evidence that the microbiota has profound effects on the development, phenotype, and function of multiple immune cell populations (25). These effects, in turn, can influence the ability to respond to antigenic challenges and susceptibility to infectious and immune-mediated diseases (26–28). Thus, variations in the mother’s microbiota can have a significant impact on the offspring’s immune capabilities.

### Effects of maternal infection on offspring immunity

Infections during pregnancy, caused by viruses, bacteria, fungi, or parasites, collectively represent a common clinical problem that can vary in severity from the relatively trivial to the life-threatening (29, 30). They can have obvious, sometimes severe, effects on the health of the fetus as well as more subtle influences on the structure and function of developing fetal tissues and organ systems. The immune system is not exempt from such developmental disturbances, and there is emerging evidence that maternal infection and, more broadly, the microbial milieu during pregnancy can have significant effects on immune function in the offspring. Each of the connections between the mother and the fetal/infant immune system can be perturbed by maternal infection or other microbial exposure, and such perturbations have the potential to alter offspring immune functions, either in the short term or more persistently. Studies in mice and humans provide evidence of these effects, as described in the sections that follow.

#### Abs

Any time a pregnant woman is infected or otherwise exposed to an antigenic stimulus, she will generate an Ab response, including Ag-specific IgG and possibly IgA. Her repertoire of circulating Abs also reflects her earlier antigenic exposures. IgG can cross the placenta into the fetal circulation, and, interestingly, recent studies in mice indicate that maternal IgG undergoes pregnancy-associated changes in glycan structure that enhance its antimicrobial capabilities in the newborn offspring (31). Secretory IgA produced by the mother is released into breast milk and transmitted to the newborn during suckling. Because the mother’s circulating IgG and breast milk IgA are largely directed against the Ags to which she has been exposed, including those derived from microbial pathogens and her own microbiota, the Abs represent vehicles for transmitting maternal immunological experience to the baby.

Transport of maternal IgG across the placenta requires the neonatal Fc receptor expressed on the syncytiotrophoblast, but other Fc receptors also probably contribute to the process (14, 32, 33). Transfer is generally proportional to serum IgG concentration (up to a point; see below). It is greatest during the third trimester of pregnancy and is affected by IgG subclass and by Ab glycosylation status (14, 15, 33, 34). Maternal infection can impair transplacental movement of IgG directed against a variety of Ags, an effect that has been documented in a large number of clinical studies, especially in the context of malaria and HIV infections (35–39). One factor contributing to this effect is the elevation of serum IgG that can occur in both malaria and HIV infection (35, 36, 38). A concentration above 15 g/L decreases the fraction of circulating IgG transported across the placenta, possibly as a result of saturation of transport mechanisms (14). But additional mechanisms are likely to be involved because maternal infection–associated decreases in transplacental Ab transfer can occur in the absence of hypergammaglobulinemia and can be selective for IgG of specific subclasses or Ag specificities (40). Maternal infection with HIV, SARS-CoV-2, and *Plasmodium falciparum* all show such subclass- and Ag-specific effects on IgG transport (40–42). This phenomenon may be explained by the changes in Ab glycosylation that are known to occur in the context of infectious and noninfectious inflammation and that can affect IgG–Fc receptor interactions (42–44).

Because maternal Abs represent an important source of protection for the first 3–6 mo of life, a significant decrease in transplacental IgG transfer can compromise antimicrobial defense in the infant (14, 15). However, transplacentally transferred maternal Abs can also interfere with the offspring’s own humoral responses, including to vaccination, either by masking vaccine Ags or by attenuating B cell activation through binding to specific Fc receptors (45, 46). It is even possible that the interaction of maternal IgG with Fc receptors on infant immune cells could influence offspring immunity more broadly. Such effects need to be considered when designing maternal immunization strategies. Finally, it is worth noting that some maternal Abs can have adverse effects on the fetus and newborn, such as Abs directed against the dengue virus or against offspring paternal Ags (14). In such situations, infection-associated impairment of IgG transport across the placenta may be beneficial.

Although IgG is generally considered to be the only isotype that is actively transported across the placenta, studies in mice indicate that maternal IgE can make its way into the fetal circulation in a complex with IgG (47, 48). The maternal IgE can bind to fetal mast cells, setting the stage for the later development of skin and airway allergic responses on exposure to the relevant Ag. A similar phenomenon may occur in humans because allergen-specific IgE can be detected in umbilical cord blood and IgE has been found in association with human fetal skin mast cells (47, 49). Whether the transplacental movement of maternal IgE is affected by maternal infection or immune activation remains to be determined.

In addition to IgG and IgE, breast milk IgA is the other maternal Ab that can influence offspring immunity. Secretory IgA, produced by maternal plasma cells in the mammary gland, is transported into breast milk by the polyimmunoglobulin receptor and exerts its effects in the gastrointestinal tract of the suckling infant (16). It provides protection against enteropathogens and plays important roles in shaping the infant’s gut microbiota. As discussed in greater detail below, microbiota composition can have significant effects on various aspects of immune function. Maternal infection and vaccination have been shown to increase the quantity of breast milk IgA specific for the infecting pathogen or vaccine, but whether these states of immune activation have a similar effect on IgA directed against other Ags is unclear (50–56). The limited data available suggest that maternal infection and other states of immune activation do not alter IgA quantity in milk (57).

#### Cytokines

Activation of a pregnant woman’s immune system as a result of infection or other stimuli is usually associated with increased expression of various inflammatory cytokines, some being produced by fetal cells, whereas others are secreted by maternal cells and then transferred across the placenta into the fetal circulation. These cytokines may influence the development and function of the fetal immune system either positively or negatively, sometimes with long-lasting consequences. There are several examples of this type of fetal immune “training” that have been observed in clinical studies and mouse experiments, and the action of specific cytokines such as type I and type II IFNs, IL-6, IL-10, and TGF-β has been implicated in some cases (58).

One of the early examples of long-term programming of the fetal immune system by maternal inflammation derives from experiments in which pregnant mice were injected with the TLR3 ligand polyinosinic:polycytidylic acid [poly(I:C)] to mimic the effects of a viral infection. In addition to behavioral traits mimicking certain aspects of autism, the adult offspring of the poly(I:C)-injected mothers displayed multiple abnormalities of the immune system, including an overall hyperinflammatory phenotype characterized by a decrease in regulatory T cells, an increase in Th17 cells, and alterations in myeloid cell populations (59). The long-lived nature of the immune abnormalities induced in offspring by this type of maternal immune activation (MIA) has focused attention on the effects of inflammation on fetal hematopoietic stem and precursor cells (HSPCs). Indeed, fetal HSPCs can directly sense inflammatory mediators, including pathogen-associated molecular patterns and various cytokines, and respond by proliferating and changing their differentiation potential (60). Because a subset of fetal HSPCs contribute to adult hematopoiesis, alterations induced in these cells by maternal inflammation can have lasting effects in the offspring (60). Illustrating such effects, the type I IFN that results from MIA with poly(I:C) in mice has been shown to lead to expansion and hyperactivation of innate-like B lymphocyte populations in the progeny, with corresponding elevation of natural Ab levels in the peritoneal cavity and increased production of IL-10 by peritoneal B-1 cells following in vitro stimulation (61). Similarly, infection of pregnant mice with *Toxoplasma gondii* led to expansion and altered differentiation potential of HSPCs in the fetal liver, with injected IFN-γ reproducing some of the effects (62). Even more strikingly, a recent (as yet unpublished) study showed that injecting pregnant mouse dams with poly(I:C) reprogrammed fetal HSPCs, leading to increased numbers and hyperresponsiveness of group 2 innate lymphoid cells in the lungs of the adult progeny (López D. A., A. Griffin, L. M. Aguilar, C.-D. Rice, E. J. Myers, K. J. Warren, R. Welner, and A. E. Beaudin, manuscript posted on bioRxiv, DOI: 10.1101/2023.11.20.567899). Moreover, the expanded and hyperresponsive group 2 innate lymphoid cells contributed to a heightened airway allergic response (D. A. López et al., manuscript posted on bioRxiv, DOI: 10.1101/2023.11.20.567899).

HSPCs are not the only fetal stem cells that are affected by maternal inflammation. When pregnant mice were infected with an attenuated strain of *Yersinia*, which was cleared quickly and never crossed the placenta, the adult offspring had increased numbers of Th17 cells in the intestinal lamina propria, making them more resistant to oral *Salmonella* infection but also more susceptible to the induction of noninfectious colitis (63). The mechanism of this effect was found to involve epigenetic reprogramming of fetal intestinal epithelial stem cells by maternal IL-6, which promoted the enhanced expression of antimicrobial genes and genes influencing Th differentiation. The results suggest that similar effects may occur in other tissues, a possibility that will no doubt be addressed in future studies.

Breast milk contains a number of cytokines that can vary widely in concentration from one individual to the next and, in some cases, with lactation stage (64–66). It is particularly rich in members of the TGF-β family, which are present in biologically relevant amounts. The cytokines may be produced by maternal leukocytes and epithelial cells, although definitive data addressing the exact source are currently lacking. Several of the cytokines, particularly TGF-β, have been shown or postulated to modulate immune function in the infant in various ways. Abnormalities in this process have been linked to the development of inflammatory diseases such as necrotizing enterocolitis, but the evidence is largely circumstantial. Maternal infection has varying effects on breast milk cytokine concentrations. In a large study of Peruvian mothers of low-birth-weight neonates, peripartum infection (unspecified febrile illness or urinary tract infection) was associated with significantly lower concentrations of some cytokines in colostrum (66). On the other hand, at least some studies of maternal HIV or SARS-CoV-2 infection have demonstrated significant elevations of breast milk TGF-β2 and other cytokines (67, 68). Whether any of these maternal infection–associated changes in breast milk cytokine levels have effects on the infant immune system remains to be clarified.

#### Ags

Ags to which a pregnant individual is exposed, from the environment or as the result of infection or vaccination, can make their way across the placenta and prime the fetal immune system (59, 69). The exact mechanisms that allow the transplacental movement of Ag are not well understood and could involve transport in the free form, as part of a complex with IgG (facilitated by neonatal Fc receptor) or in association with cells or microvesicles. The details of how Ag is presented to and sensed by fetal adaptive immune cells are also not clear. Regardless, both clinical studies and experiments in mice have provided evidence, in the form of newborn Ag-specific Abs or T cell responses, that lymphocytes can be sensitized by prenatal exposure to Ag, including in the context of several types of maternal infection (58, 69). Such sensitization can result in lymphocyte activation or tolerance, depending on circumstances, with corresponding effects on the offspring’s response when subsequently exposed to the Ag. Placental malaria infection, where malaria Ags gain access to the fetal circulation, is a valuable model to examine pathogen-specific fetal immune responses (70–73). For instance, in utero exposure to malaria Ags, as occurs in a significant fraction of pregnancies in endemic regions of Africa, has been reported to induce either a protective effector CD4^+^T cell response or a suppressive T-regulatory cell response, based on factors such as timing of exposure, relative fetal immune maturation, and the presence of active placental infection (74). The offspring have increased malaria resistance or susceptibility, respectively, at least for the first few years after birth (70).

#### Microbiota

Studies in humans and mice carried out over the last couple of decades have provided evidence of the profound influence of the microbiota on the development and function of the immune system and on the occurrence of infectious and immune-mediated diseases (25–28). The maternal microbiome during pregnancy can also have significant impacts on immune system programming (75). The newborn’s microbiota is initially derived from the mother during birth, a process that represents a major mechanism mediating maternal influence on offspring immunity (23, 24). Plausible links can be made between maternal infection or immune activation, abnormalities of the mother’s and newborn’s microbiota, and infant immune function, but studies that have directly examined this chain of causality are hard to find. However, several investigations in both humans and mice have shown that antibiotic treatment of the mother during pregnancy, either presumed or documented to alter the microbiota composition, can affect the offspring’s immune capabilities or risk of infection and immune-mediated disease. For instance, a large birth cohort study from Denmark showed that children born to mothers treated with antibiotics during pregnancy had an elevated risk of infection-related hospitalization and that the risk increased with the number of antibiotics used and use close to birth (76). A recent meta-analysis of 31 clinical and animal studies supported this finding and extended the increased risk of pregnancy antibiotics to asthma and atopic dermatitis (77).

#### Cells

There are limited reports on the presence and functionality of maternal microchimeric cells (MMCs) in human infants, especially in relation to maternal infection and offspring disease susceptibility. High numbers of maternal memory T cells have been demonstrated in cord blood (78). In one study of a nested cohort in Tanzania, inflammatory placental malaria infection was associated with increased levels of cord blood MMCs that correlated with decreased symptomatic malaria in infected infants (79). This observation was similar to another study in American children that found the presence of MMCs in whole blood to be protective from later development of asthma (80). In a study of a Ugandan cohort, although *P. falciparum* parasitemia in the first half of pregnancy was also associated with increased prevalence of MMCs in cord blood, there was no association between MMC numbers in preparasitemia samples and first parasitemia samples in childhood, suggesting that acute infection may not alter the levels of microchimerism (81). Furthermore, although the malaria studies found an association between maternal infectious state and presence of MMCs in offspring, an analysis of a South African maternal–infant cohort found that HIV-exposed but uninfected children had fewer MMCs than HIV-unexposed children, and this correlated with an altered response to bacillus Calmette-Guérin vaccination (82). One explanation for these discordant observations might be the different levels of MIA and placental inflammation across cohorts that might contribute to altered MMC functional imprinting and trafficking.

In a study using an animal model of preconception infection with OVA-expressing *Listeria*, OVA-specific maternal T cells were shown to be transferred to the fetus during pregnancy (83). The presence of these chimeric T cells in offspring correlated with less severe disease following infectious challenge in early life. Maternal Ag-primed T cells may also be transferred through breastmilk, and in an animal model of preconceptual helminth infection, maternal CD4^+^ Th2 cells acquired from breast milk protected from the same nematode challenge in both early and later life (84). MMCs also protected from a neonatal CMV infection by promoting the differentiation of myeloid cells in the murine fetal bone marrow, although this was not studied in the context of maternal infection (85). MIA resulting from infectious challenge can also impact fetal brain development and long-term neurobehavioral processes (86). Viral infections, including rubella (87, 88) and influenza (89, 90), as well as bacterial infections, such as urinary tract infections (91, 92), during pregnancy are associated with autism spectrum disorder and other neurodevelopmental disorders (93). Although a role for MMCs was never considered in these human studies, MMCs have been detected in the offspring brain, and one study in animals reported that alterations in their number led to dysregulated offspring behaviors, although this was also not studied in the context of maternal inflammation (94).

### Vaccinations and pregnancy

Early life represents a period of dramatic escalation in antigenic encounters by the relatively naive and still developing newborn immune system. It is not unexpected, therefore, that infectious morbidity and mortality are highest in the first weeks after birth (95). Maternal immunization during pregnancy represents a valuable strategy for protecting both the mother and her offspring from various infectious diseases. In addition to transfer of passive immunity to offspring, maternal vaccination has been associated with reduced rates of maternal morbidity and mortality, as well as improved birth outcomes, including reduced risks of preterm birth and low birth weight. We discuss these findings in the context of four vaccines currently approved for pregnant women: tetanus, diphtheria, acellular pertussis (Tdap), inactivated influenza, SARS-CoV-2, and respiratory syncytial virus (RSV).

#### Tdap vaccine

Pertussis, a highly contagious respiratory illness caused by *Bordetella pertussis*, is a significant public health concern, particularly for newborns who are too young to be vaccinated themselves. In 2011, the Tdap vaccine was recommended for pregnant women, to be administered at the beginning of the third trimester when transfer of IgG Ab via the placenta is at its highest. High titers of pertussis-specific IgG were detected in both the vaccinated mother and her newborn offspring (96). Ag-specific IgA was also detected in the colostrum of vaccinated mothers and in the newborns’ gut (97). Maternal Tdap vaccination during pregnancy was >90% effective in protecting against pertussis during the first 2 mo of life (98, 99). However, it was noted that Tdap vaccines do not fully protect the mother from infection (100). Furthermore, maternal Abs were linked to decreases in the newborn’s own immune response to subsequent pertussis vaccination as well as to heterologous polio and pneumococcus vaccine Ags (101–104). There is limited information on the impact of pregnancy-associated Tdap vaccination on cellular immune responses in offspring after pertussis primary and booster vaccination. One study found robust induction of Th1, Th2, and Th17 responses after acellular pertussis vaccination in infants (105), whereas another reported significant changes in the phenotype and cytokine responsiveness of infant PBMCs to *B. pertussis* as well as acellular pertussis vaccine Ags (106). Whether there is a direct impact of maternal Tdap vaccination on the developing human fetal adaptive immune system is not known. However, early studies in tetanus toxoid–vaccinated pregnant women identified toxoid-specific IgM Ab in cord blood, indicating fetal B cell sensitization (maternal IgM cannot cross the placenta) (107, 108). The impact of Tdap vaccination during pregnancy on the maternal microbiome as well as on the acquisition of MMCs by the offspring is entirely unknown.

#### Inactivated influenza vaccine

Influenza infection during pregnancy is associated with an increased risk of adverse maternal and neonatal outcomes, including preterm birth, low birth weight, neonatal death, and maternal complications such as pneumonia and respiratory failure (109). Both the inactivated and live influenza vaccines are currently not indicated for children below 6 mo of age. Thus, the quadrivalent inactivated influenza vaccine is recommended for all pregnant women, regardless of pregnancy trimester (110). However, flu vaccine administered in the late second or third trimester offers significant benefits to both mother and infant by providing humoral immunity that lasts 6–7 mo after vaccination and that is transferable to offspring across the placenta and via breast milk (111). A blunting effect of flu-specific maternal Abs on offspring responses has not been observed in humans (104). In a murine model of pregnancy-associated influenza immunization, high maternal Ab titers in offspring did not prevent neonatal B cell activation (112). Rather, the blunting effect was observed in the inability of germinal center B cells to become plasma and memory B cells. Thus, high titers of maternal Ab shaped the germinal center output and the Ag-specific B cell repertoire in murine offspring. Priming of the fetal immune system by maternal flu vaccination has been reported. Influenza-specific IgM Abs were detected in the cord blood of infants of immunized mothers (113). Using MHC tetramers to identify Ag-specific T cells, one study demonstrated the presence of memory T cells in the cord blood following vaccination against influenza during pregnancy (114). As with the Tdap vaccine, effects of flu vaccination on the maternal microbiome or the microchiome have not been investigated.

#### SARS-CoV-2 vaccine

The emergence of the COVID-19 pandemic, caused by the novel SARS-CoV-2 virus, has posed significant challenges to global public health, with pregnant women being particularly vulnerable because of potential complications associated with viral infections (115, 116). COVID-19 vaccines are recommended for pregnant women and have been shown to be effective in reducing hospitalization and poor outcomes after COVID-19 infection in infants (117). The mRNA vaccine elicits a robust Ag-specific Ab response that is detected in cord blood, breast milk, and serum of infants of vaccinated mothers (118–120). Of interest, unlike the flu and Tdap vaccines, mRNA-based COVID-19 vaccination during pregnancy did not elicit an IgM response in cord blood (121). There was also an absence of Spike mRNA or Ag in the placenta and cord blood, suggesting a lack of fetal priming in utero with this mode of vaccination (121, 122). COVID-19 vaccines have recently been approved for infants aged 6 mo and older. There are, however, as yet no published reports describing a blunting response to the vaccine after maternal COVID-19 immunization. The impact of maternal COVID-19 vaccine on the microbiome and MMCs is also unexplored.

#### RSV vaccine

RSV infection is a significant contributor to respiratory morbidity and mortality among infants, particularly those born prematurely or with underlying health conditions. Given the lack of effective treatments for RSV and the limitations of passive immunoprophylaxis with mAbs, maternal immunization has emerged as a promising approach to protect vulnerable infants from severe RSV-related illness. Recent data from clinical trials show that the RSVpreF Ag vaccine is safe and efficacious for use in pregnant women and prevents severe RSV disease in infants. Maternal RSV vaccine reduced the risk of severe RSV disease by 82% within 3 mo and by 69% within 6 mo after birth. The precise nature of the Ab response to RSV Ag in pregnant vaccinated women, as well as the titers achieved in offspring, are yet to be determined. Activation of fetal immunity by the vaccine also needs to be investigated.

## Conclusions and Future Research Priorities

The study of transgenerational effects on immune function is relatively new, particularly in relation to humans. Some of the specific gaps in knowledge in this area have been alluded to in preceding sections. More broadly speaking, the field has thus far focused largely on the role of maternal Abs (transplacentally transferred IgG, breast milk IgA) and, more recently, on the maternal microbiota. Much less work has been done on other components of the maternal immune system that might influence offspring immunity: cells, cytokines, and additional soluble molecules. For instance, there is a significant paucity of information on MMCs and their effects in the fetus and infant. The same holds true for the multiple cytokines that are produced by maternal immune cells in the context of infection or other inflammatory stimulus and that have the potential to cross the placenta. Even with respect to transplacental transfer of IgG, the mechanisms involved in the selective impairment of transport of certain Ab subclasses or specificities that is seen in some maternal infections are not fully understood. We also do not have much insight into the effects of maternal infection on breast milk IgA. Recent advances in single-cell technologies and systems immunology, as well as increased access to human developmental tissue, will undoubtedly lead to important biological insights into these early-life processes (123).

Another area that has not received much attention and that is worth exploring is the sex specificity of maternal effects on fetal and infant immunity and how that might affect imprinting of offspring immune function by the maternal microbial environment. The bidirectional interaction between mother and fetus is influenced by the sex of the latter, both because of sex-specific differences in fetal physiology, including the physiology of the immune system, and because the mother responds differently to a male versus female fetus (124). Clinical studies have demonstrated that the sex of the fetus affects obstetric outcomes, including the occurrence of complications such as gestational diabetes and pregnancy-induced hypertension (125, 126). Whether fetal sex is a factor in imprinting of the developing immune system by maternal microbial exposure and the molecular mechanisms involved are issues that are yet to be clarified.

The inclusion of pregnant women in clinical trials evaluating vaccine safety and efficacy has been historically limited, leading to gaps in knowledge regarding the optimal vaccination strategies for this population. Vaccination during pregnancy confers numerous benefits to the mother and her offspring. However, high levels of vaccine-induced maternally derived Abs can blunt the infant’s own response to vaccination. Strategies to bypass these “blunting” effects need to be explored and may include the use of different adjuvants as well as altering the route of vaccination. There is also a paucity of information on the nonspecific effects (NSEs) of vaccines administered during pregnancy. Live vaccines are typically associated with beneficial NSEs on all-cause childhood mortality, but nonlive vaccines can have detrimental NSEs (127). Although live vaccines are currently not recommended during pregnancy, nonlive vaccines such as the influenza H1N1 vaccine have been shown to enhance susceptibility to unrelated infections in females (128). The consequences of maternal innate immune activation triggered by vaccines during pregnancy on offspring immunity remains unexplored.

The maternal influence on offspring immunity represents a potential therapeutic opportunity. It raises the possibility that manipulating the mother’s environmental exposures during pregnancy might be a way to program the fetal/neonatal immune system to modify susceptibility to infection or other types of disease. Pregnancy vaccination is one example of such an intervention, but one could envision extending the idea to other approaches, such as the administration of specific probiotics or other microbial products. The development and implementation of such strategies to “educate” early-life immunity could have significant long-term benefits for individual and public health.
